# Synergizing Off-Target Predictions for In Silico Insights of CENH3 Knockout in Cannabis through CRISPR/Cas

**DOI:** 10.3390/molecules26072053

**Published:** 2021-04-03

**Authors:** Mohsen Hesami, Mohsen Yoosefzadeh Najafabadi, Kristian Adamek, Davoud Torkamaneh, Andrew Maxwell Phineas Jones

**Affiliations:** 1 Department of Plant Agriculture, University of Guelph, Guelph, ON N1G 2W1, Canada; mhesami@uoguelph.ca (M.H.); myoosefz@uoguelph.ca (M.Y.N.); kadamek@uoguelph.ca (K.A.); dtorkama@uoguelph.ca (D.T.); 2 Département de Phytologie, Université Laval, Québec City, QC G1V 0A6, Canada

**Keywords:** hemp, marijuana, machine learning algorithm, ensemble model, CENH3, sgRNA, genome editing, MIT score, CFD score

## Abstract

The clustered regularly interspaced short palindromic repeats (CRISPR)/Cas-mediated genome editing system has recently been used for haploid production in plants. Haploid induction using the CRISPR/Cas system represents an attractive approach in cannabis, an economically important industrial, recreational, and medicinal plant. However, the CRISPR system requires the design of precise (on-target) single-guide RNA (sgRNA). Therefore, it is essential to predict off-target activity of the designed sgRNAs to avoid unexpected outcomes. The current study is aimed to assess the predictive ability of three machine learning (ML) algorithms (radial basis function (RBF), support vector machine (SVM), and random forest (RF)) alongside the ensemble-bagging (E-B) strategy by synergizing MIT and cutting frequency determination (CFD) scores to predict sgRNA off-target activity through in silico targeting a histone H3-like centromeric protein, HTR12, in cannabis. The RF algorithm exhibited the highest precision, recall, and F-measure compared to all the tested individual algorithms with values of 0.61, 0.64, and 0.62, respectively. We then used the RF algorithm as a meta-classifier for the E-B method, which led to an increased precision with an F-measure of 0.62 and 0.66, respectively. The E-B algorithm had the highest area under the precision recall curves (AUC-PRC; 0.74) and area under the receiver operating characteristic (ROC) curves (AUC-ROC; 0.71), displaying the success of using E-B as one of the common ensemble strategies. This study constitutes a foundational resource of utilizing ML models to predict gRNA off-target activities in cannabis.

## 1. Introduction

*Cannabis sativa* L. has a long history of human use for various applications including fibers, food, medicine, and for its psychoactive properties [1]. The crop is generally divided and regulated as two main groups based on the level of produced tetrahydrocannabinol (THC), with anything below 0.3% THC considered hemp and plants that produce 0.3% THC or more classified as marijuana [2].

Marijuana and some hemp genotypes are dioecious crops meaning the male and female reproductive systems occur on separate plants [3]. For cannabinoid production, seedless and unfertilized female cannabis flowers are the most economical product [4]. Due to these challenges, breeding strategies in cannabis are complicated, and the existing cultivars are not genetically or phenotypically uniform and plants are most often propagated using clonal methods. While developing a true F1 hybrid seed would overcome this challenge and offer a more efficient propagation strategy, producing inbred lines for F1 hybrid seed production through self-pollination is difficult due to the dioecious nature [4]. This can be overcome by inducing hermaphroditic plants to facilitate self-pollination, but this takes time and is thought to lead to increased hermaphroditism in the offspring. Therefore, there is a need for new breeding strategies to overcome these bottlenecks and rapidly produce homozygous breeding lines.

While hemp can also be used for the production and isolation of non-psychoactive cannabinoids (e.g., cannabidiol (CBD), cannabigerol (CBG), etc.), the existing cultivars are ill-suited for this application [2]. Since there was previously no commercial use for non-psychoactive cannabinoids and it is critical that THC levels remain below the threshold (below 0.3% in most countries), breeders have tended to select plants that produce relatively low levels of cannabinoids in general, typically between 1–3% [5], compared to an average THC content of 17.1% in modern medical/recreational genetics [4]. Further, for cannabinoid production, growers prefer using dioecious cultivars (separate male and female plants) to produce unfertilized female plants to avoid formation of the seed and maximize cannabinoid content [4]. Currently, many of the existing hemp cultivars are monoecious (male and female flowers produced on the same plant) and are generally not suitable for cannabinoid production [6]. As such, there is a significant need for new hemp varieties that are suited for outdoor production (i.e., dioecious), contain high levels of non-psychoactive cannabinoids (e.g., CBD), and reliably remain under the 0.3% THC threshold [2].

One of the challenges in developing new hemp varieties to produce high levels of non-psychoactive cannabinoids is that as the cannabinoid pathway is promoted, the likelihood of exceeding the 0.3% THC limit increases [4]. Screening of the existing industrial hemp lines [7] showed that 43% of the 167 cultivars exceeded the 0.3% limit for THC [8]. This challenge becomes more acute when developing cultivars for high cannabinoid content since even with high CBD:THC ratios, this limit can easily be exceeded. In a more recent field trial of hemp specifically developed for cannabinoid production, only seven of the 30 tested cultivars remained equal to or below 0.3% THC, and they generally produced less CBD than the rest (below 8%) [8]. Further, due to the open pollination breeding platforms typically used for hemp, there remains significant genetic and chemical variability among plants within a cultivar, making it more difficult to consistently remain below 0.3% THC [4]. In order to develop new varieties with high levels of non-psychoactive cannabinoids while ensuring THC is reliably below 0.3%, breeding strategies that provide a higher degree of genetic and phenotypic uniformity are required [2].

The shift from highly variable open breeding platforms to more controlled production of the genetically uniform F1 hybrid seed has happened in many other species through repeated inbreeding or double-haploid induction [9]. Among the first examples, prior to the 1930s, corn (*Zea mays*) was an open pollinated crop that displayed a high degree of variation but has since transitioned to the F1 hybrid seed that is over 99% genetically uniform [10]. In addition to greater uniformity, this transition has resulted in increased yields, stress tolerance, and overall productivity [11]. In the case of cannabis, transitioning to F1 hybrid seed production might lead to similar achievements as well as help to ensure a consistently low level of THC to meet regulatory requirements.

The prerequisite to developing a genetically uniform F1 hybrid seed is the production of inbred lines through repeated self-pollination to obtain nearly homozygous parental lines [12]. This process is challenging in cannabis as it is a dioecious plant meaning the male and female reproductive systems occur on separate plants. Researchers have developed methods to overcome this challenge by applying various chemical compounds, such as gibberellic acid, silver nitrate, or silver thiosulphate, to induce male flowers on female plants [11]. This method facilitates the production of inbred lines as well as of feminized seeds (genetically female seeds). Feminized seeds are ideal for hemp cultivation to produce cannabinoids. While this approach provides an interesting solution to overcome the dioecy in cannabis plants, it is time-consuming and there is a belief that repeated artificial induction of male flowers results in an increased rate of hermaphroditism in the offspring [11,12]. Although the increased prevalence of hermaphroditism has yet to be evaluated, it is theoretically plausible through epigenetic mechanisms [9].

Another approach to producing inbred lines is through androgenesis, using either microspore or anther culture techniques [12]. In this process, the development of microspores is redirected from pollen toward somatic embryogenesis to produce a haploid plant. Once a haploid plant is produced, it is treated with an antimitotic agent (e.g., colchicine and oryzalin) to produce a completely homozygous diploid plant [11]. However, this process is technically challenging, is often highly genotype-specific, and has not yet been developed for cannabis.

To overcome some of the challenges associated with androgenesis, directed engineering of centromeric histone H3 (CENH3) genes has been used to interfere with centromere activity and induce haploid seed production [13]. Recently, direct modification of CENH3 through the clustered regularly interspaced short palindromic repeats with Cas9 (CRISPR/Cas9) system has been used to produce haploid plants in recalcitrant crops such as maize [14,15] and wheat [13]. The merit of this approach over conventional in vitro culture-based methods is that once an inducer line is generated, it can be employed to induce haploidy in other genetic backgrounds, simplifying the process and negating the development of in vitro culture protocols for each genotype [14,15]. While this approach has not yet been developed for cannabis, it is particularly attractive as cannabis is a relatively recalcitrant species and organogenesis protocols have not yet been successful.

The CRISPR/Cas system is a reliable and efficient method for accurate genome editing [13,16]. Within the CRISPR/Cas-mediated genome editing system, the endonuclease-mediated Cas is guided to the targeted gene by the single guide RNA (sgRNA) [17]. If the sgRNA matches with the targeted gene correctly, the Cas9 endonuclease can precisely edit the gene [18]. However, unexpected mutations can be caused by the predesigned sgRNA and a protospacer adjacent motif (PAM). Recent studies report that off-target mutations can be tackled by precise sgRNA design [17]. Several studies [18,19,20] also document that off-target mutations can be universal and should be considered in CRISPR/Cas studies. In most cases, the off-target sites are homologous with one or more mismatches to the on-target sites [18]. Generally, there are four mismatch categories to classify on-target and off-target sites based on sequence characteristics: (i) while genomic sequences have the same length and are correctly matched, the PAM is mismatched; (ii) although there are nucleotide mismatches, genomic sequences have the same length; (iii) genomic sequences have various lengths and there are some additional nucleotide bases; (iv) genomic sequences have various lengths and there are some missing nucleotide bases [21,22]. Therefore, it is necessary to assess the off-target activity to increase the reliability and accuracy of the CRISPR/Cas9 systems.

To minimize off-target mutations, several studies investigated genome-wide DNA damage induced by CRISPR/Cas9 through genomic profiling methods [23,24,25]. Such studies provide experimental findings to develop computational methodologies such as Cas-OFFinder [26], CasOT [27], CRISPRseek [28], and CRISPRdirect [29] for studying and forecasting potential off-target sites. Recent studies [18,19,20] have approved the reliability of using machine learning algorithms to predict the sgRNA cleavage efficiency in non-plant species. The current study compares multiple machine learning algorithms to maximize off-target prediction for eventual use in producing a cannabis haploid inducer line for rapid and efficient production of double haploids. While cannabis is relatively recalcitrant, there are some regeneration systems developed [30,31,32], so producing the inducer line should be possible.

## 2. Results

We used *Cannabis sativa* histone H3-like centromeric protein HTR12 as a candidate for the CRISPR/Cas9 system. To predict the sgRNA cleavage efficiency, an initial dataset of 1900 putative off-target sequences including 950 true positive off-targets identified with a mismatch count of up to four recognized by CRISPR [33] was used. We implemented three algorithms including random forest (RF), radial basis function (RBF), and support vector machine (SVM). Then, MIT and CFD scores were used as input variables for a comparative analysis of individual algorithms. We also performed combined prediction using the ensemble-bagging (E-B) algorithm with the predictions for all the three models.

**Figure 1:**
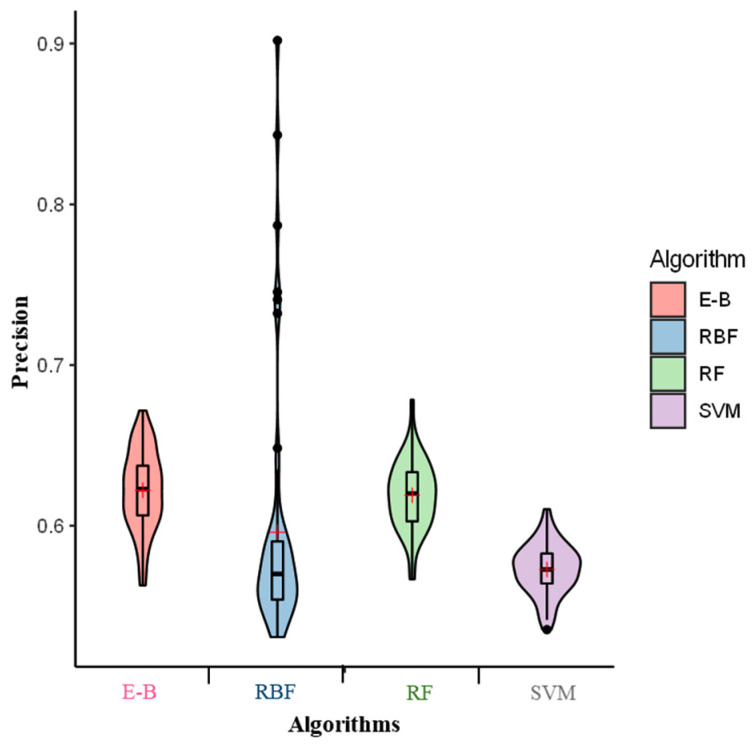
Precision of the radial Basis function (RBF), support vector machine (SVM), random forest (RF), and ensemble-bagging (E-B) algorithms for off-target activity prediction using MIT and CDF scores. × indicates the mean performance of precision.

As can be seen in Figure 1, the RF, RBF, and SVM had the highest to lowest precision (i.e., 0.61, 0.60, and 0.57, respectively) to predict off-target activity. The ensemble model through the bagging method (E-B) consistently outperformed all the individual algorithms with a precision value of 0.62 indicating a better and persistent prediction performance.

**Figure 2:**
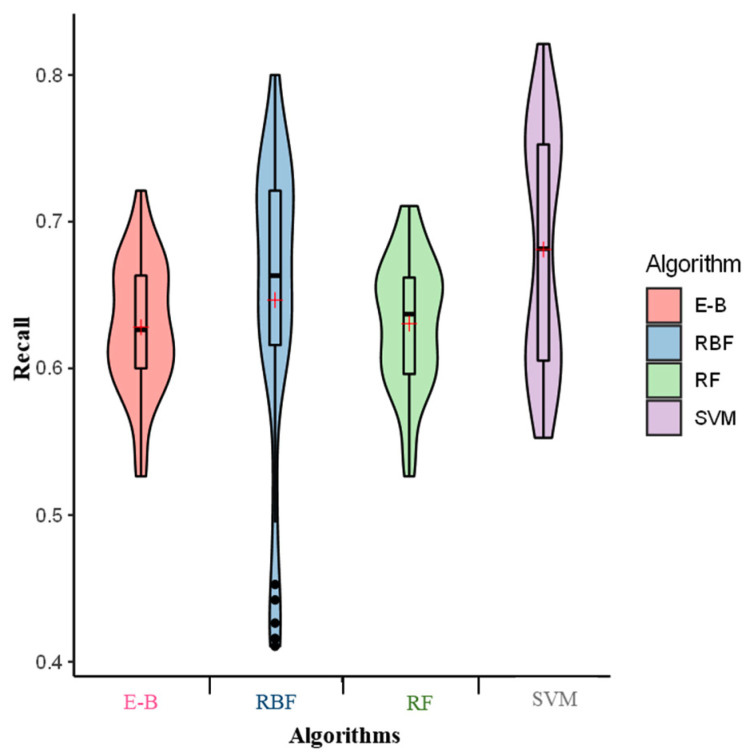
Recall of the radial basis function (RBF), support vector machine (SVM), random forest (RF), and ensemble-bagging (E-B) algorithms for off-target activity prediction using MIT and CDF scores. × indicates the mean performance of recall.

We then classified different models based on a recall value. In this scenario, the SVM demonstrated the highest average recall value (0.67) over all the tested individual and ensemble algorithms. The RBF and RF were placed second and third with an average recall value of 0.65 and 0.64, respectively (Figure 2).

**Figure 3:**
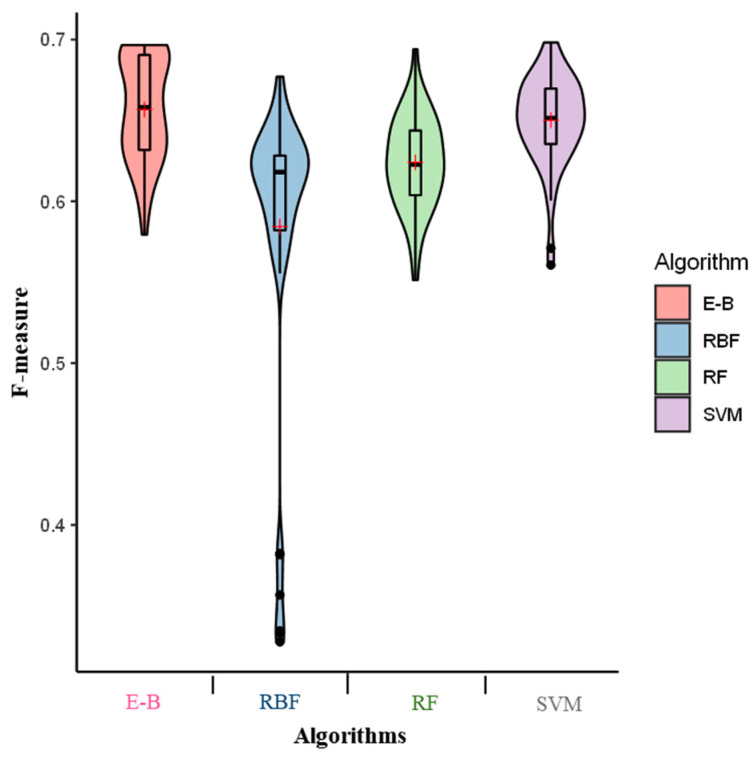
F-measure of the radial basis function (RBF), support vector machine (SVM), random forest (RF), and ensemble-bagging (E-B) algorithms for off-target activity prediction using MIT and CDF scores. × indicates the mean performance of F-measure.

The E-B model exhibited the lowest average recall value (0.63) among all the tested algorithms. To capture both properties of precision and recall into a single measure, an F-measure was estimated for all the tested algorithms [19]. Our result indicated the highest value of F-measure for the E-B model (0.66). However, the F-measures of SVM, RF, and RBF amounted to 0.65, 0.62, and 0.58, respectively (Figure 3).

**Figure 4:**
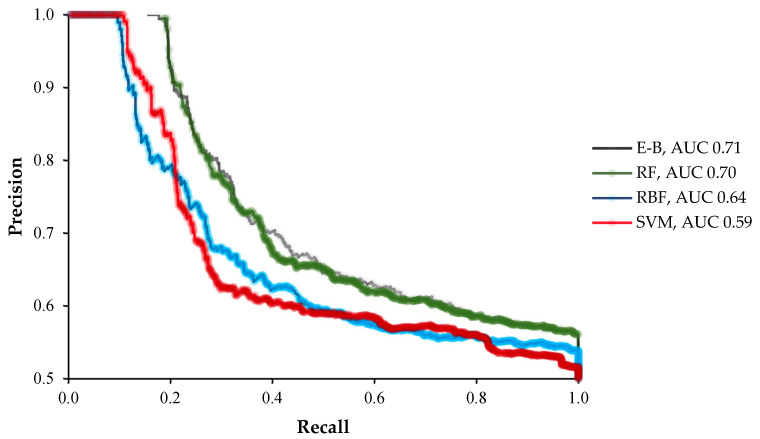
The area under the precision recall curves (PRC; AUC-PRC) of the radial basis function (RBF), support vector machine (SVM), random forest (RF), and ensemble-bagging (E-B) algorithms for off-target activity prediction using MIT and CDF scores.

**Figure 5:**
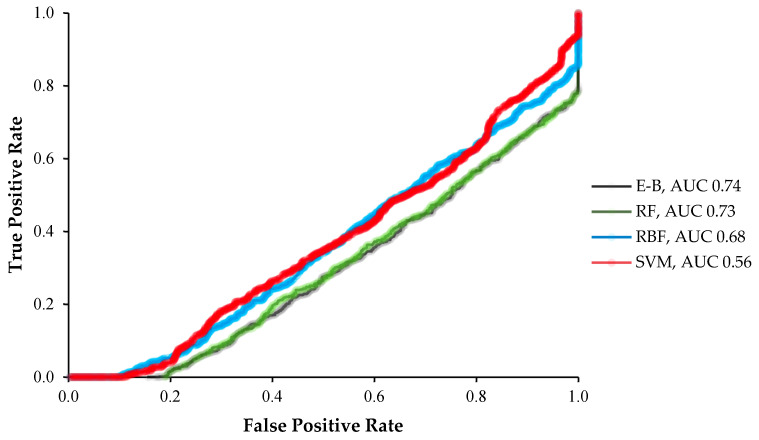
The area under the receiver operating characteristic (ROC) curves (AUC-ROC) of the radial basis function (RBF), support vector machine (SVM), random forest (RF), and ensemble-bagging (E-B) algorithms for off-target activity prediction using MIT and CDF scores.

In terms of the comparative analysis values for all the tested machine learning algorithms, the E-B model outperformed all the individual algorithms both in the area under the precision recall curve (AUC-PRC) and the area under receiver operating characteristic curve (AUC-ROC) with the values of 0.74 and 0.71, respectively (Figure 4 and Figure 5). RF and RBF were placed the second and third with the AUC-PRC of 0.70 and 0.64 and the AUC-ROC of 0.73 and 0.68, respectively (Figure 4 and Figure 5). The lowest prediction performance based on the AUC-PRC and AUC-ROC value was found for the SVM algorithm (Figure 4 and Figure 5).

## 3. Discussion

To date, multiple methods such as direct in vitro culture (e.g., androgenesis and gynogenesis), selective hybridization through intraspecific and interspecific crosses, and genome editing (i.e., CIRSPR-Cas9) using CENH3 genes have been used to produce haploid plants [11]. Haploid production is a powerful method in plant breeding and genetic engineering when chromosome doubling is used to produce completely homozygous double haploid lines much quicker than traditional production of inbred lines through repeated self-pollination [34,35]. Recently, haploid plants have been produced by direct modification of CENH3 via the CRISPR/Cas9 system in recalcitrant crops such as maize [14,15] and wheat [13]. Although there is no report on using the CRISPR/Cas9 system to produce haploid plants in cannabis, it represents a potentially reliable and powerful method to produce haploid plants in cannabis. The advantage of this approach over traditional culture-based methods is that once an inducer line is produced, it can be used to induce haploidy in other genetic backgrounds, negating the optimization of culture conditions for each genotype [14,15]. However, the first step of using the CRISPR/Cas9 system is to design a precise sgRNA with minimal off-target activities [36].

The selection of optimal sgRNAs with low, ideally no off-target and high on-target activity is an important prerequisite to perform CRISPR-mediated genome editing [36]. The development of web-based tools for in silico sgRNA designing, such as CRISPOR [33] and algorithms for forecasting sgRNA activity, has considerably facilitated the improvement of a CRISPR-mediated genome editing system [18]. Compared to the prediction of CRISPR efficiency, a precise and accurate forecasting of off-target activity is the most challenging step in conducting a CRISPR-mediated genome editing experiment [18]. Consequently, uninvertible and potential off-target activity is the most critical issue that limits the practical application of CRISPR [37]. Indeed, tolerating the minimum mismatches between the off-target site and the sgRNA spacer can be considered as one of the main reasons of off-targets in the CRISPR system [22]. Additionally, previous studies [38,39] documented that the off-target sites of CRISPR are not random.

To date, two main steps are usually used to study and quantify the off-target activities: (i) in silico detection of off-target sites through various webtools such as CRISPOR [33] and (ii) scoring based on selection and ranking such as MIT [40] and CFD [39]. For instance, the off-target score assessed by MIT is based on the nucleotide numbers of mismatches and their distances. This can be then applied to classify whether the off-target score surpasses the threshold (i.e., cut-off value) of 66 [41]. The CRISPOR recommends the MIT score as an off-target reference. MIT can summarize all important off-target sequences and achieve high accuracy through applying an aggregation score of a single gRNA [33]. Consequently, the off-target score is predicted through a CFD (cutting frequency determination) method by multiplying the base frequency in each gRNA spacer sequence position [39]. Therefore, the performance of sgRNA specificity in CFD depends on the number, position, and composition of mismatches between the target DNA and sgRNA sequences [39].

Recently, the application of machine learning algorithms has been successfully investigated in different areas such as genome editing [18,19,20,42], prediction of transcription factor target genes [43,44], phenomics [45,46,47], and plant tissue culture [46,48,49,50,51]. Conventional statistical methods such as ANOVA and simple regression methods are typically recommended for small datasets with limited dimensions [48]. One of the major impediments of using conventional statistical methods is high probability of overfitting [38]. To overcome this obstacle, ML algorithms can be employed [19]. To the best of our knowledge, machine learning algorithms have never been used to predict the sgRNA off-target activity in cannabis and in general in plants. In this study, we found that the RF algorithm shows the highest precision, recall, and F-measure, indicating the highest prediction performance among all the tested individual algorithms. In the RF algorithm, trees are trained based on multiple random subsamples of the original dataset [48]. This enables the RF algorithm to generate stable and better prediction for new data lines not necessary existing in the training dataset [52]. The successful use of RF has been reported in different areas of plant science [43,48,52].

In general, three types of prediction error including bias, variance, and irreducible error (noise) are reported in application of individual ML algorithms. [53]. Therefore, ensemble algorithms were built to improve robustness over a single model with combining the predictions of several models [54,55]. In this study, the predictions derived from the RF, RBF, and SVM algorithms were used to build an ensemble model based on the bagging method. For selecting the classifier for the bagging method, RF was selected owing to its highest prediction accuracy. This resulted in achieving the highest precision recall and F-measure using the E-B model. Although the number of off-target sequences in each chromosome had a similar distribution, using individual ML algorithms might be subject to some levels of bias and overfitting. Therefore, ensemble algorithms can be considered as a reliable strategy to overcome this problem. In this study, the E-B algorithm outperformed all individual ML algorithms with the highest level of F-measure. F-measure is known as a reliable parameter that can be used to evaluate efficiency and accuracy of ML algorithms [19]. Recent studies have reported the success of using stochastic gradient boosting and E-B modeling in plant science [19,55], but not in the computational component of the plant genome editing. The E-B model exhibited the highest off-target prediction performance (AUC-ROC of 0.74 and AUC-PRC of 0.71) based on MIT and CFD scores. These high AUC-ROC and AUC-PRC scores using the E-B model provided a promising prediction ability in non-determined circumstances which non-existent instances added to the original dataset [18,19].

Cannabis is generally recalcitrant and in vitro culture response is very genotype-dependent [3], making the development of double haploids very challenging in general. The developed method in this study offers an alternative, but still requires the production of an inducer line through plant regeneration. However, once a single inducer line is produced, it can then be used across a wide range of genetic backgrounds and eliminate the need for developing microspore culture techniques for each genotype. Given that there are reports of plant regeneration in cannabis [30,31,32], developing the inducer line should be possible and provide a significant advantage over other approaches.

## 4. Materials and Methods

### 4.1. Datasets

*Cannabis sativa* histone H3-like centromeric protein HTR12 was used for predicting off-target activities in cannabis. To identify this gene in cannabis, CENH3 (GenBank ID GU166737.1) in *Brassica rapa* was used to blast. In the current study, the CRISPOR [33] online tool was used to recognize off-target sequences with a mismatched number of up to four. The dataset contained 950 off-target (positive) sequences which were labeled 1, and others were negative (non-off-targets). Two scoring approaches (MIT [40] and CFD [21]) were applied to score each putative off-target sequence according to the locations, identities, and mismatched number between sgRNA and DNA. The CFD score established by Doench et al. [21] that determines the cutting frequency and calculates the potential off-target of DNA–sgRNA intersections was used. In the CFD approach, the number, identity, and position of mismatches between the target DNA and sgRNA play pivotal roles in discovering activity [21]. The MIT score [40] evaluates and calculates the potential off-target sequences by considering a weight per position of mismatch between the target DNA and sgRNA. These scores were used as the features of classifiers. Examples of sgRNAs, putative off-target DNA sequences, their genomic coordinates, CFD and MIT scores are presented in Table 1.

The target variable (output) of models is a value between 0 and 1, demonstrating the off-target effect probability. The score of 1 demonstrates a perfect match for the off-target effect, while the score of 0 shows the opposite.

### 4.2. Classification Models

In this study, four machine learning algorithms including random forest (RF) [48], radial basis function (RBF) [56], support vector machine (SVM) [57], and the ensemble model using the bagging method (E-B) [54] were used to study and predict off-target activity. The results of each algorithm were individually evaluated and then an algorithm with the highest prediction performance was selected as a meta-classifier for the E-B algorithm. The Weka software version 3.9.4 [58] was used to analyze all the machine learning algorithms.

### 4.3. Evaluation Criteria

**Figure 6:**
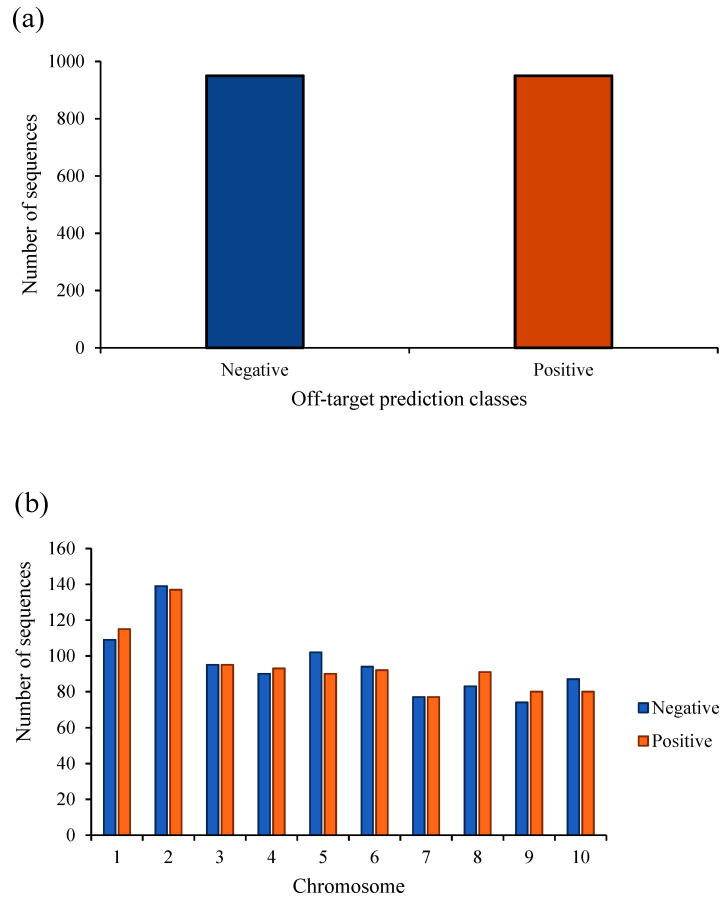
The distribution of off-target prediction classes in (**a**) the whole database and (**b**) each chromosome with respect to CFD and MIT scores.

The abovementioned machine learning algorithms were implemented using the initial dataset based on a five-fold cross-validation procedure [59] with ten repetitions since the target classes were completely balanced (Figure 6).

In order to evaluate the prediction performance of each algorithm, the values of recall (Equation (1)), precision (Equation (2)), and F-Measure (Equation (3)) for the validation dataset were estimated via the following formulas:

(1) $$\mathrm{Recall}=\frac{\mathrm{TP}}{\mathrm{TP}+\mathrm{FN}}$$

(2) $$\mathrm{Precision}=\frac{\mathrm{TP}}{\mathrm{TP}+\mathrm{FP}}$$

(3) $$F-\mathrm{Measure}\;=2\times \frac{\mathrm{Precision}\times \mathrm{Recall}}{\mathrm{Precision}+\mathrm{Recall}}$$

where TP is the number of true positives, FP is the number of false positives, and FN stands for the number of false negatives.

For a better interpretation of results, the area under the precision recall curves (PRC; AUC-PRC [19]) was estimated based on the precision and recall values. Furthermore, the area under the receiver operating characteristic (ROC) curves (AUC-ROC) was evaluated based on the true positive rate and false positive rate values. PRC and ROC are complementary components [19].

## 5. Conclusions

One of the objectives of this study was to determine the best machine learning algorithm for predicting the off-target activity in cannabis. By using MIT and CFD scores as inputs, the RF overperformed all the individual algorithms. Therefore, this algorithm was chosen as a meta-classifier for constructing the E-B model. E-B, one of the ensemble strategies, showed the highest precision, F-measure, AUC-PRC, and AUC-ROC accuracy compared to the individual machine learning algorithms. Therefore, E-B was recommended as an appropriate and reliable model for predicting the off-target activity in cannabis. It is expected that in the near future, consistent, comprehensive, and sequencing-based datasets with high quality and efficiency will be generated. Therefore, continuous efforts are needed to enhance the precision of sgRNA design with low off-target and high on-target activities. With the expansion of the data volume from the CRISPR usage and a deeper understanding of the CRISPR system to be discovered, learning-based sgRNA methods would increase the prediction of off-target activities to meet the requirements for application of CRISPR-mediated genome editing. However, this study was an in silico work and needs to be validated. Before using the CRISPR system in cannabis, it is necessary to develop a reliable regeneration protocol. We are currently developing regeneration methods to validate the approach and establish such an inducer line.

## Figures and Tables

**Table 1:** Examples of sgRNA, DNA, the coordinates of DNA, MIT and CFD scores.

sgRNA	Putative Off-Target DNA Sequences	Chromosome	Start	End	MIT	CFD
TTAGCAGTGTCCAAGTCTTCTGG	TCAGCAGCGTCTAAATCTTCAGG	7	638	660	0.199	0.434
TTAGCAGTGTCCAAGTCTTCTGG	CTAGAGGTGTCCATGTCTTCAGG	5	21,767	21,789	0.135	0.187
AGCTTTAGTTGCACTTCAGGAGG	AGCTTTAATTGAATTTCATGGGG	8	2079	2101	0.033	0.349
CACGTCGACTTGGAGGGAAAGGG	CAGGTCGACGTCGAGGAAAAAGG	3	3689	3711	0.259	0.123
AGCCTGGAACAAAGGCTCTCCGG	AGACTGCAACAAAGCATCTCCGG	5	1624	1646	0.047	0.162

## Data Availability

Not applicable.
